# Characterization and Drug Sensitivity of a New High-Grade Myxofibrosarcoma Cell Line

**DOI:** 10.3390/cells7110186

**Published:** 2018-10-25

**Authors:** Giacomo Miserocchi, Alessandro De Vita, Laura Mercatali, Federica Recine, Chiara Liverani, Chiara Spadazzi, Federica Pieri, Nada Riva, Alberto Bongiovanni, Roberto Casadei, Valentina Fausti, Toni Ibrahim

**Affiliations:** 1 Osteoncology and Rare Tumors Center, Istituto Scientifico Romagnolo per lo Studio e la Cura dei Tumori (IRST) IRCCS, Via P. Maroncelli 40, 47014 Meldola, Italy; giacomo.miserocchi@irst.emr.it (G.M.); alessandro.devita@irst.emr.it (A.D.V.); federica.recine@irst.emr.it (F.R.); chiara.liverani@irst.emr.it (C.L.); chiara.spadazzi@irst.emr.it (C.S.); nada.riva@irst.emr.it (N.R.); alberto.bongiovanni@irst.emr.it (A.B.); valentina.fausti@irst.emr.it (V.F.); toni.ibrahim@irst.emr.it (T.I.); 2 Pathology Unit, Morgagni-Pierantoni Hospital, Via Carlo Forlanini 34, 47100 Forlì, Italy; federica.pieri@auslromagna.it; 3 Orthopedic Unit, AUSL della Romagna -Forlì, Via Carlo Forlanini 34, 47100 Forlì, Italy; roberto.casadei@auslromagna.it

**Keywords:** myxofibrosarcoma, primary culture, CD109, cell line establishment

## Abstract

Myxofibrosarcoma (MFS) belongs to the group of sarcoma tumors, which represent only 1% of the totality of adult tumors worldwide. Thus, given the rare nature of this cancer, this makes the availability of MFS cell lines difficult. In an attempt to partially fill this gap, we immortalized a primary culture of MFS (IM-MFS-1) and compared the cell morphology with patient’s tumor tissue. IM-MFS-1 was genetically characterized through a Comparative Genomic Hybridization (CGH) array and the mesenchymal phenotype was evaluated using Polymerase chain reaction (PCR) and immunofluorescence staining. Drug sensitivity for MFS therapies was monitored over time in cultures. We confirmed the conservation of the patient’s tumor cell morphology and of the mesenchymal phenotype. Conversely, the synthesis and expression of CD109, a TGFβ co-receptor used to facilitate the diagnosis of high-grade MFS diagnosis, was maintained constant until high cancer cell line passages. The CGH array revealed a complex karyotype with cytogenetic alterations that include chromosome regions associated with genes involved in tumor processes. Cytotoxicity assays show drug sensitivity constantly increased during the culture passages until a plateau was reached. In conclusion, we established and characterized a new MFS cell line that can be used for future preclinical and molecular studies on soft tissue sarcomas.

## 1. Introduction

Myxofibrosarcoma (MFS) represents one of the most common soft tissue sarcomas usually occurring in the limbs of older patients [1]. MFS lesions, previously grouped as malignant fibrous histiocytoma (MFH) variants, are now described by the World Health Organization (WHO) as lesions with the presence of myxoid background and recognized as a distinct pathologic entity [2]. This fibroblastic tumor is characterized by a highly heterogeneous histological appearance, including pleomorphism and curvilinear vessels, which often delays diagnosis [2,3,4,5]. Surgery in association with adjuvant or neoadjuvant radiotherapy is the standard treatment for patients with localized disease [6]. Although MFS has a better prognosis than other sarcoma histotypes [1,7], between 50% and 60% of patients develop local recurrences [8,9]. The metastatic potential of MFS is related to the grade of the lesion; low-grade lesions show multiple local recurrences but have a low metastatic rate, whereas high-grade lesions are associated with a higher metastatic capacity. However, MFS has a significantly lower metastatic potential than pleomorphic sarcomas [2,6,7,8,9,10,11]. 

Chemotherapy represents the standard treatment in the metastatic setting, but the outcome is generally poor [9,12]. Patients with advanced soft tissue sarcomas are normally treated with a combination of anthracyclines and ifosfamide as front-line therapy. In this setting, previously untreated patients show a response rate of 20–30% that decreases to 10% in a second-line setting [13]. Nevertheless, the role of adjuvant and neo-adjuvant chemotherapy and radiotherapy requires further evaluation [14]. The low incidence of sarcomas and the consequently insufficient patient enrolment are the main reasons for the low number of drugs currently used in this clinical setting. From a preclinical point of view, the difficulty in studying MFS stems from the paucity of commercially available cell lines and the resultant lack of in vitro and in vivo models [15]. The presence of different tumor subclones in the same lesion is one of the reasons for the resistance to anti-cancer agents [16]. The establishment of cell lines partially maintains the subclone sets and thus represents an opportunity to enrich the number of MFS cell lines available for preclinical research.

The present study describes our morphological, molecular and genetic characterization of a new MFS cell line, IM-MFS-1, obtained from a local recurrence in the right knee of a patient. We also report on the changes in drug sensitivity observed during culture with first- and second-line treatments until a stabilization of the response rate was obtained.

## 2. Materials and Methods

### 2.1. Patient History

The primary culture was obtained from a surgically resected local recurrence of myxofibrosarcoma, characterized by a pleomorphic appearance, in the right knee of a 69-year-old male patient. Fluorescence in situ hybridization (FISH) was negative for MDM2, thus excluding a diagnosis of well-differentiated/dedifferentiated liposarcoma and confirming the MFS nature of the lesion [17]. The first radical surgery was performed in December 2013 after diagnosis of high-grade myxofibrosarcoma. A local recurrence was detected after 3 months (March 2014) and the patient underwent re-surgery, with complete excision of the lesion. The patient remained disease-free for 10 months when he developed a second local recurrence characterized by a subcutaneous nodule of 3.8 cm. The mass was surgically removed, histological examination revealing necrotic and myxoid areas with the presence of pleomorphic cells, and the patient underwent adjuvant radiotherapy. This study was approved by the IRST-Area Vasta Romagna Ethics Committee, approval no. 4751/2015, and performed according to Good Clinical Practice standards and the Declaration of Helsinki. The patient provided written informed consent to take part in the study. 

### 2.2. Cell Culture Procedure

The mass was surgically excised, carefully analyzed by an experienced sarcoma pathologist, and transported under sterile conditions to the Biosciences Laboratory of our institute within 3 hours of removal. The tumor sample, as previously reported [18], was washed twice in phosphate buffered saline (PBS) and minced with a surgical scalpel into fragments of about 0.5–1 mm^3^. The fragments were enzymatically digested with 2 mg/mL collagenase type I (Millipore Corporation, Billerica, MA, USA) 1:1 in Dulbecco’s-modified Eagle’s medium (DMEM, Invitrogen, Darmstadt, Germany) for 15 min at 37 °C under stirring conditions. The sample was then stored overnight in agitation at room temperature. The digestion process was stopped by adding DMEM supplemented with 10% fetal bovine medium (Invitrogen), 1% penicillin/streptomycin and 1% glutamine. The suspension obtained was filtered with a 100-µm sterile mesh filter (CellTrics, Partec, Münster, Germany) to remove non-digested residue. Cells were seeded in a monolayer culture and maintained at 37 °C in a 5% CO_2_ humidified atmosphere. They were cultured for about 24 months and trypsinized once every 3 days.

### 2.3. Cell Proliferation Assay

IM-MFS-1 cells were seeded in 96-well plates at two different concentrations (5 × 10^3^ and 10 × 10^3^ cells/well) and cultured in 200 µL of D-MEM. Cell proliferation was analyzed by MTT assay, according to the protocol previously elsewhere [19]. Doubling times were calculated through growth curves.

### 2.4. Immunohistochemical and Immunofluorescence Staining

Cells were stained with hematoxylin and eosin (H&E), desmin, αSMA, S100, phalloidin, vimentin and Cytokeratin Pan (pan-CK) to evaluate the morphological features and mesenchymal phenotype of the culture. H&E staining of the patient’s tissue sample was performed on formalin-fixed paraffin-embedded sections (FFPE) at the Pathology Unit of Morgagni-Pierantoni Hospital (Forlì). H&E staining of the primary culture was carried out using 5-µm-thick slides of a 3D collagen-based scaffold included in paraffin in a cryomold (25 mm × 20 mm × 5 mm). The collagen-based scaffolds were synthesized as previously described [20]. Sectioned slides were hydrated and stained with hematoxylin (Sigma-Aldrich, Saint Louis, MO, USA) and eosin (Sigma-Aldrich) for 15 min and 30 sec, respectively, and then analyzed under the microscope (Axioskop, Carl Zeiss, Gottïngen, Germany). Desmin, αSMA and S100 immunohistochemical staining were performed on 5-µm-thick slides of a 3D collagen-based scaffold at the Pathology Unit of Morgagni-Pierantoni Hospital (Forlì) using the standard diagnostic protocols.

Pan-CK immunohistochemical staining was performed on cells spun onto glass slides using the Shandon CytoSpin III Cytocentrifuge. Epithelial cell detection was performed using the murine monoclonal antibody (mAb) A45-B/B3 (specific for a common epitope of Cytokeratin polypeptides) of the Epimet kit (Micromet, Munich, Germany). The protocol includes permeabilization by a kit detergent (5 min), fixation using a formaldehyde-based solution (10 min), conjugation of the mAb to the cytoskeletal CK (45 min in a humid and dark environment) and the use of a specific conjugate to bind the mAb. The method includes a 2 min counterstaining with hematoxylin. Immunofluorescence staining was performed on cells seeded in 4-well CorningTM FalconTM chambered cell culture slides at a concentration of 5 × 10^4^ cells/well. Cells were washed twice with phosphate-buffered saline (PBS) and then fixed for 10 min in 3.7% paraformaldehyde (Polyscience, Niles, IL, USA). Cells were rehydrated and permeabilized in 0.1% Triton X-100 for 5 min. After washing twice in PBS, the samples were incubated, covered with phalloidin stock solution (Life Technologies, Foster City, CA, USA) and diluted 1:20 or with vimentin (BD Biosciences, San Jose, CA, USA) in PBS for 10 min at room temperature. β catenin staining was performed using β catenin Ab (Santa Cruz, Dallas, TX, USA) diluted 1:400 in PBS and Alexa Fluor® 488 goat-rabbit IgG (Thermo Scientific) as secondary Ab. The wells were washed with PBS after nuclei counterstaining with 4′,6-diamidino-2-phenylindole (DAPI) (Invitrogen, Life Technologies). Imaging was performed with an inverted fluorescence microscope. 

### 2.5. Genome Array Comparative Genomic Hybridization (aCGH)

aCGH was performed on DNA extracted from tumor cells at passage 50. QIACube (Qiagen, Hilden, Germany) was used to obtain DNA, in accordance with the manufacturer’s recommendations. The assay was performed using the 180 K platform CGH microarray (Agilent Technologies, Santa Clara, CA, USA). The processes of labeling and hybridization were performed following the manufacturer’s instructions (Agilent Technologies). Gender-matched DNA was used as CGH-array control (Agilent Technologies). Samples were analyzed by Agilent scanner (G2505C) and Agilent CytoGenomics 3.0 Feature Extraction for CytoGenomics (Agilent Technologies). Data analysis was performed by Agilent Cytogenomics software v.4.0.3.12 (Agilent Technologies) using an aberration filter of minimum probe number ≥5 and an absolute log ratio ≥0.15.

### 2.6. Gene Expression Analysis

mRNA extraction was performed on trypsinized cell specimens conserved at −80 °C in TRIzol Reagent (Invitrogen, Carlsbad, CA, USA). cDNA was obtained by reverse transcribing 500 ng of RNA using iScript cDNA Synthesis Kit (BioRad, Hercules, CA, USA). A total of 2 µL of cDNA was added to 18 µL of 2x Taqman Universal PCR Master Mix (Applied Biosystems, Foster City, CA, USA). Amplification was performed by Real-Time polymerase chain reaction (PCR) assay using 7500 Real-Time PCR System (Applied Biosystems). The following genes were analyzed: *EGFR*, *JAG1, vimentin*, *CTNNB1*, *SPARC*, *TGFβ*, *CD109*, *MMP2* and *ITGA10. ACTB* and *HPRT* were used as housekeeping genes. The obtained data were normalized to the housekeeping genes with the delta-delta Ct (2^−ΔΔCt^) method. 

### 2.7. Drugs Sensitivity Test

Drug sensitivity analysis was performed by seeding 1 × 10^4^ cells/well in 96-well plates. After 2 days, the cells were treated with plasmatic peak concentrations of epirubicin (EPI) and trabectedin (TRABE), in accordance with the pharmacokinetic/clinical data for each drug. EPI was administered at a concentration of 2 µg/mL [21,22,23] and TRABE at 2.2 × 10^–5^ µm [24,25]. After a 72 h exposure, survival assays were performed using the MTT test (Sigma-Aldrich) following the manufacturer’s protocol [26]. 

### 2.8. DNA Fragmentation Detection

DNA fragmentation generated during the apoptosis process was detected by the terminal deoxynucleotidyl transferase (TdT) nick and labeling (TUNEL) assay. Cultures at passage 1 and 50 were seeded at a concentration of 1 × 10^4^ cells/well in 96-well plates and exposed to the same drug concentrations used in the drug sensitivity test for 3 days. At the end of treatment, cells were washed twice in PBS, incubated in 1% paraformaldehyde for 15 min on ice and later in 70% ice-cold ethanol for 1 h. After two washes in PBS, the cells were permeabilized in 0.1% Triton X-100 in PBS for 5 min and exposed to a TdT and Fluorescein isothiocyanate (FITC) conjugated dUTP deoxynucleotides 1:1 solution (Roche Diagnostic GmbH, Mannheim, Germany) at 37 °C for 90 min in a dark humidified environment. Counterstaining was performed with ProLong Gold antifade reagent with DAPI for nuclei detection. Samples were analyzed using an inverted fluorescence microscopy.

### 2.9. Statistical Analysis

Each experiment was repeated at least 3 times (8 technical replicates for each condition were performed in the drug sensitivity tests). Data are shown as mean ± standard deviation (SD), or mean ± standard error (SE), as stated, with *n* indicating the number of replicates. The two-tailed Student’s *t*-test was used to define the differences between groups and *p* values < 0.05 were considered significant. 

## 3. Results

### 3.1. Establishment of IM-MFS-1 Myxofibrosarcoma Cell Line

**Figure 1:**
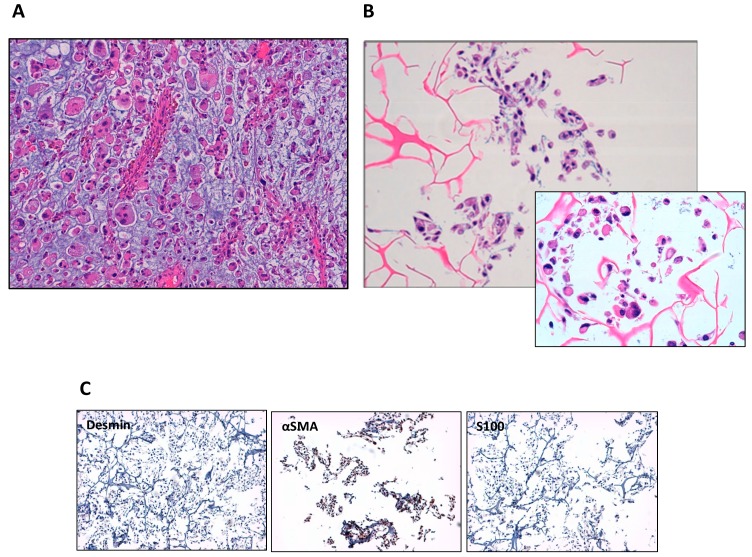
Morphologic comparison between the patient’s tumor tissue and primary culture. H&E staining of the patient’s tumor tissue. The image shows high-grade myxofibrosarcoma cells and the myxoid matrix (light-blue stroma) at 20× magnification (**A**). H&E staining of the patient-derived primary culture. Some of the morphologic features of the tissue of origin are maintained, i.e., the presence of giant cells, prominent nuclei and disseminated vacuoles at 20× and 40× magnification (**B**). Immunohistochemical staining for desmin, αSMA and S100 on IM-MFS-1 at passage 1 at 20× magnification (**C**).

The patient’s tumor tissue was mechanically and enzymatically digested to obtain a single cell suspension and seeded on monolayer plates. Over the next days, the cells were cultured successfully to 80–90% confluence. In order to compare the morphology of the primary culture with that of the patient’s tissue, we seeded the cells on a 3D collagen-based scaffold, which provides a more faithful representation of cell population morphology than monolayer surfaces [27]. After H&E staining, the images were studied by an expert pathologist who noted important similarities between the tissue and primary culture (Figure 1A,B). The former showed curvilinear vessels, pleomorphic neoplastic cells and an infiltrating myxoid component, all features typical of epithelioid myxofibrosarcomas [2]. Many of the cell morphology features were conserved, in particular giant cells, prominent nuclei and disseminated vacuoles. Moreover, the lack of an MFS-specific biomarker makes this culture system essential for the correct identification of a malignant phenotype. Immunohistochemical analyses of desmin, αSMA and S100 were performed on IM-MFS-1 samples grown in 3D scaffolds at passages 1 and 50 (Figure 1C and Supplementary Figure S1). The culture was positive for αSMA and negative for desmin and S100 in both passages.

**Figure 2:**
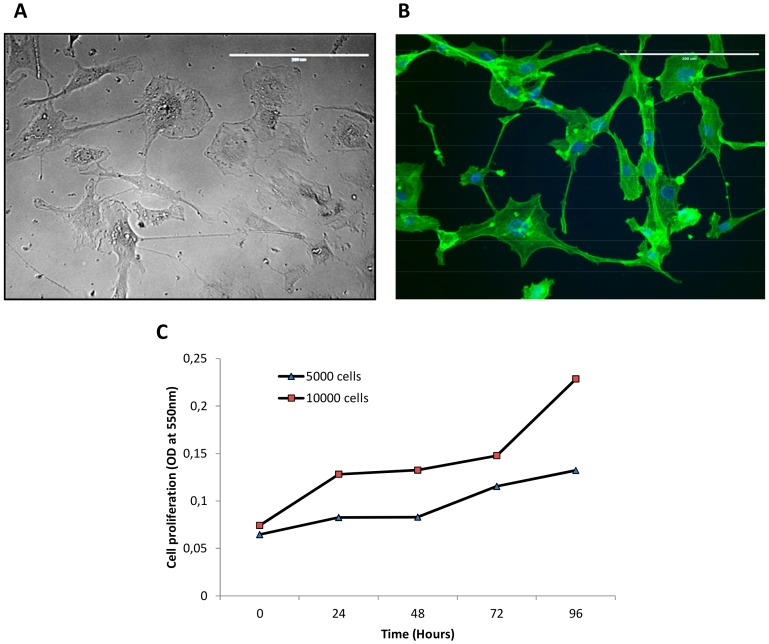
Morphologic and proliferation characterization of IM-MFS-1 cell line. A 40× image of IM-FMS-1 cell line at passage 50. Scale bar 200 µm (**A**). Immunofluorescence phalloidin staining showing the cell line morphology on a 2D monolayer support. Nuclei were counterstained with DAPI. Scale bar 200 µm (**B**). Proliferation analysis shows low growth rate for both conditions of 5 × 10^3^ and 10 × 10^3^ cells/well (Optical density [OD] read at 550 nm) (**C**).

The culture was maintained for 120 passages (at the time of publication) and the morphology did not change significantly over time. During the long-term cultivation (passage 50), the cells appeared voluminous and showed abundant cytoplasm and prominent nuclei (Figure 2A). Phalloidin immunofluorescence staining was performed to display several offshoots produced by the cells in 2D monolayer culture (Figure 2B). The culture doubling time was measured at concentrations of 5 × 10^3^ and 10 × 10^3^ cells/well (Figure 2C). Both the conditions showed a relatively low growth rate, with a double time occurred in 72 h.

### 3.2. Mesenchymal Phenotype, β Catenin Distribution and CD109 Positivity of IM-MFS-1 

**Figure 3:**
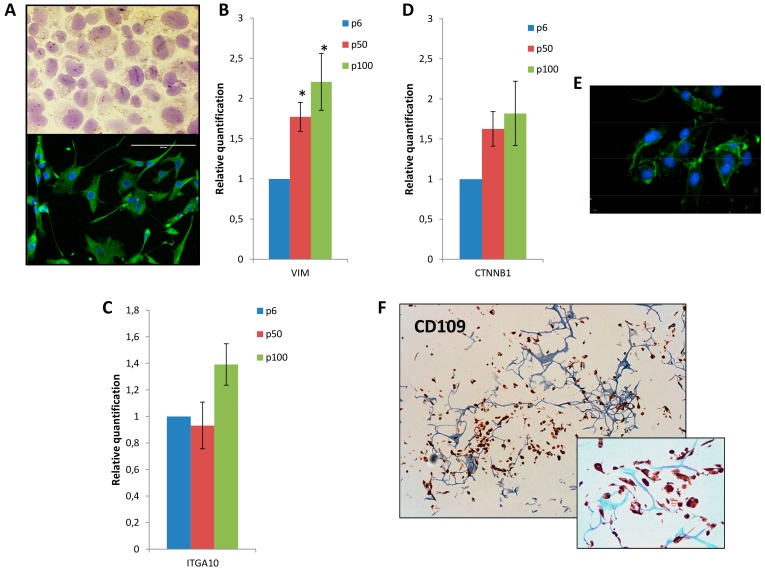
Mesenchymal phenotype, β catenin distribution and CD109 positivity of IM-MFS-1 cell line. Immunohistochemical staining shows negativity for the epithelial marker pan-CK, whereas, vimentin immunofluorescence staining revealed uniform positivity of IM-MFS-1 cells at passage 50. 40× magnification (**A**). Relative quantification of gene expression of mesenchymal markers vimentin. During the time in culture, the marker expression gradually increased from passage 6 (p6) to passages 50 (p50) and 100 (p100). Values are normalized to passage 6. In particular, vimentin showed a fold change of 0.77 and 1.21 at p50 and p100 respectively. (*) At *p* ˂ 0.05 (**B**) Relative quantification of *ITGA10* gene expression at different time points. Similar levels of mRNA were observed at passages 6 and 50, while an increase in the expression of the marker was seen at passage 100, with a fold change of 0.39. Values are normalized to passage 6 (**C**). Relative quantification of *CTNNB1*. During the time in culture *CTNNB1* expression increased constantly, with a fold change of 0.63 (p50) and 0.82 (p100), respectively. Values are normalized to passage 6. (**D**). Distribution of β catenin. Immunofluorescence staining revealed a cytoplasmic accumulation of β catenin protein at 40× magnification (**E**). Immunohistochemical staining for CD109 at p50 revealed a heterogeneous positivity of IM-MFS-1 cells at 20× and 40× magnification (**F**).

We performed pan-CK and vimentin staining to evaluate the mesenchymal phenotype of our established cell line, IM-MFS-1. Cells were negative for the epithelial marker and strongly positive for the mesenchymal one (Figure 3A). We confirmed the presence of a mesenchymal phenotype by evaluating the expression of vimentin and TGFβ. PCR arrays performed on samples at three different time-points of the culture, i.e., at passages 6, 50 and 100, revealed vimentin expression increased over time (Figure 3B), which is consistent with a high-grade MFS phenotype [1,2]. We then assessed the expression of genes related to migration and proliferation processes. Although IM-MFS-1 was positive for all the genes tested, no significant differences in the levels of *EGFR, JAG1, SPARC, CD109* and *MMP2* were seen between low and high passages (Supplementary Figure S2). We also evaluated the expression status of *ITGA10*, a gene that encodes for integrin-α10 which is known to be involved in MFS progression [28]. IM-MFS-1 expressed ITGA10 in the early passages and the mRNA quantification had increased at passage 100 (Figure 3C).

β catenin, a protein encoded by the *CTNNB1* gene, plays a role in physiological homeostasis. It has been demonstrated that an aberrant high expression of β catenin is present in several cancers [29]. During the time in culture, IM-MFS-1 showed an increased expression of *CTNNB1* (Figure 3D). Immunofluorescence analysis revealed a cytoplasmic distribution of β catenin in IM-MFS-1 cells (Figure 3E).

Finally, we investigated the expression of CD109, a TGFβ co-receptor located on the surface of endothelial cells, a subpopulation of CD34+ bone marrow cells, platelets and activated T-lymphocytes [30]. This glycoprotein regulates the degradation and endocytosis of TGFβ, inhibiting downstream signaling [31]. IM-MFS-1 cells conserved a strong positivity to CD109 from passages 6 to 50 (Figure 3F). This result confirms the maintenance of CD109 overexpression during the culture passages and demonstrates the positivity of IM-MFS-1 for this receptor.

### 3.3. IM-MFS-1 Cytogenetic Evaluation

**Figure 4:**
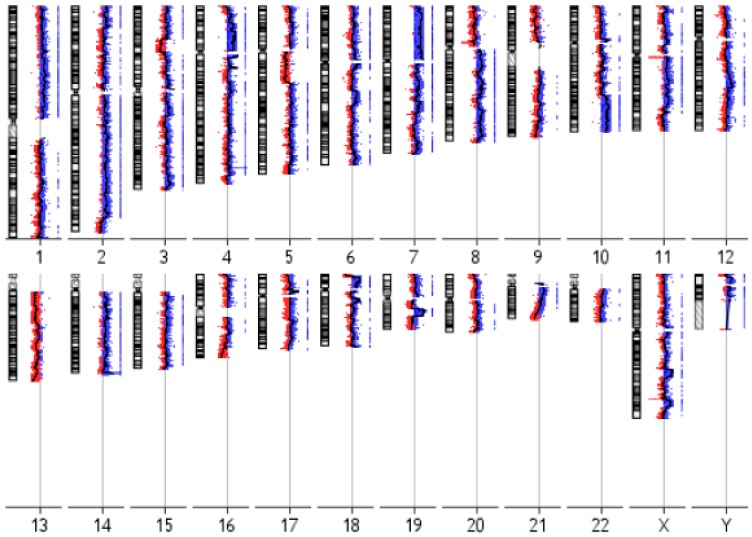
CGH profile of IM-MFS-1. Overview of chromosomal cytogenetic alterations in IM-MFS-1 cell line at passage 50. Gain and amplification alterations are shown in blue, while loss and deletions are shown in red.

IM-MFS-1 at passage 50 was cytogenetically characterized using the aCGH technique, the cells showing a complex molecular karyotype with a high number of cytogenetic alterations (Figure 4). The gain and loss aberrations located in cytogenetic regions that contain candidate genes involved in cancer processes are shown in Table 1. The CGH array revealed the presence of gain alterations in chromosome 7 regions, in particular: 7q21.11-q21.13, 7q21.11, q21.12, 7q21.13-q21.2, 7q21.3 and 7q22.1. Gains at 7q21.1-22.1 regions are frequently occurring aberrations in MFS [32,33]. Our array showed alterations in 7p21.1-p22.1 and 7q33-q35, including the 7q34 portion containing the *BRAF* gene sequence. This proto-oncogene is implicated in cell growth signaling cascades, and its dysfunction may be associated with cancerogenesis. RB1 is an inhibitor of cell cycle progression encoded in 13q10~q31 region. The CGH array showed a genomic loss of 13q14.2, suggesting a dysfunction of RB1 in IM-MFS-1 cell line, as often detected in MFS [33]. Several 19p+ abnormalities potentially correlated with unfavorable clinical outcome in MFS were also detected [34].

### 3.4. Increased Drug Sensitivity of IM-MFS-1

**Figure 5:**
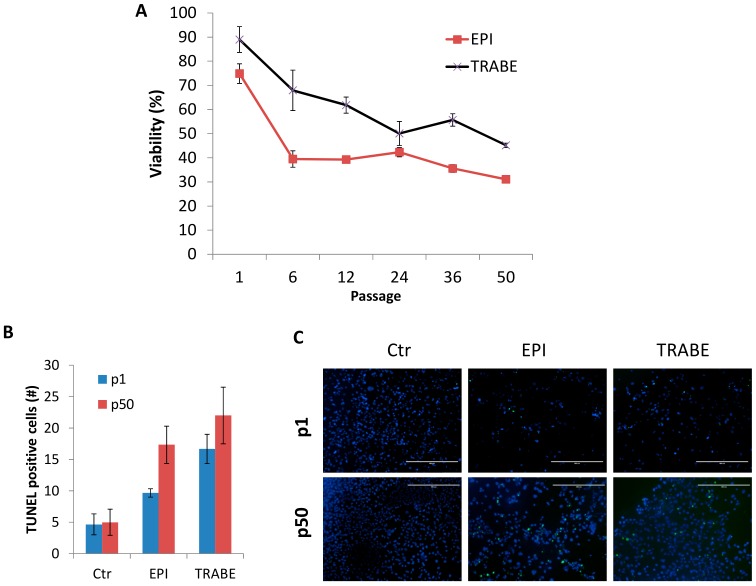
Sensitivity of primary culture and IM-MFS-1 cell line to chemotherapy treatments during the culture period. Survival percentage of IM-MFS-1 cells at different passages treated with epirubicin (EPI) and trabectedin (TRABE). All treatments reduced cell viability at each subsequent passage until a plateau was reached. Treatments were compared with the control (Ctr) conditions at each passage (**A**). Number of TUNEL-positive cells in primary culture (p1) and IM-MFS-1 and cell line at p50. Data were analyzed with Image J software (NIH Image, Bethesda, MD, USA). (**B**) TUNEL staining of primary culture (p1) and IM-MFS-1 cell line at passage 50 (p50) for treated and untreated (Ctr) conditions. Green, TUNEL-positive cells; blue, nuclei counterstained with DAPI (**C**).

The sensitivity of IM-MFS-1 cell line to two different chemotherapeutic treatments was regularly tested during passages 1 to 50 (Figure 5A). At passage 1 both treatments induced a modest effect on the primary culture, with viability rates of 74.87% for EPI and 88.97% for TRABE. Over time, cell sensitivity increased to each drug. The activity of the treatments increased at each passage until an efficacy plateau was reached. At passage 50, the percentages of viability were 31.07% for EPI and 45.18% for TRABE. Treatment induced apoptosis measured at passages 1 and 50 confirmed the above findings (Figure 5B,C).

## 4. Discussion

Myxofibrosarcoma is the most common sarcoma of the elderly, frequently occurring in the extremities [1,2,3]. Around 50–60% of all cases have multiple local recurrences [1,8,9,35]. MFS lesions are histologically characterized by prominent myxoid stroma and spindle and pleomorphic cells with abundant eosinophilic cytoplasm and atypical nuclei [2,36]. A substantial limitation for preclinical research into MFS is the paucity of cell lines. In fact, very few studies to date have been carried out on the establishment of MFS long-term cultures from surgical specimens [15,33,37]. Lohberger et al. described their morphological and genomic characterization of two different subclones of the same MFS lesion, while Kawashima et al. reported on the heterogenic morphology of a new cell line and of xenograft-derived tumors in mice [33,37,38]. The establishment of new MFS cell lines has thus become a priority. 

IM-MFS-1 is a new cell line established from a primary culture of a local relapse of high-grade MFS with pleomorphic features occurring in a 69-year-old patient. Immediately after tissue digestion, the culture showed the presence of different subclones characterized by the typical morphological features of MFS lesions. The comparison between the patient’s malignant tissue and the primary culture seeded on a 3D collagen-based scaffold confirmed the conservation of the malignant morphology. The 3D device was used because it represents an optimal model for the reproduction of morphological cell features [26,27,39]. This aspect was extremely important for determining the malignancy of the cell population and for discriminating between tumor and healthy cell components. A sarcoma pathologist expert confirmed the presence of 100% cancer cells. 

The successful acquisition of an immortalized phenotype and the capacity to grow in monolayer supports are not always predictable during the establishment process. Salawu et al. obtained seven soft tissue sarcoma cell lines from a starting group of 47 primary cultures [15]. In fact, during the in vitro phase, the destiny of cell cultures may vary from early senescence to loss of growth at low passages to the achievement of a long-term culture. We maintained IM-MFS-1 in long-term culture for 100 passages, demonstrating the stabilization of the growth of the cell population. Immunohistological staining showed cell positivity for αSMA and negativity for desmin and S100 at passages 1 and 50, indicating no change in the synthesis of the markers during the period in culture. When seeded in 2D monolayer cultures, IM-MFS-1 showed multiple prominent sprouting and different morphological features, as shown by phalloidin staining. The conservation of a heterogenic morphology further confirmed the isolation of several malignant subclones from the patient’s tissue and excluded the possibility of selecting a single clone population. Thus, IM-MFS-1 is composed of only cancer cells that reflect the heterogeneity and morphology of the patient’s cancer cell population. 

As the use of plastic monolayer cultures often causes genetic drifts during the time in culture [40], we decided to investigate the gene expression of several markers involved in cancer processes at different culture passages. No significant differences were observed in genes related to cell migration and proliferation (*EGFR, JAG1, SPARC, HRAS, CD109* and *MMP2*) did not show significant differences between a low and high number of passages. We also assessed the conservation of IM-MFS-1 stem-like phenotypes during culture time because, like all soft tissue sarcomas, they are of mesenchymal origin. In particular, we analyzed the gene expression and synthesis of vimentin [36]. The marker showed a trend of enhanced expression over time in culture, demonstrating that standard monocultures promoted the occurrence of a stem-like phenotype. In fact, at high passages, cells continued to be positive for vimentin. Lohberger et al. obtained similar results on the established MFS MUG-Myx1 cell line [38]. Both IM-MFS-1 and MUG-Myx1 cell morphology did not change during the time in culture, indeed both cell lines were characterized by prominent nucleoli and abundant cytoplasm, and the mesenchymal marker vimentin remained strongly positive. MUG-Myx1 has a higher proliferation rate with a doubling time of 24 h with respect to the 72 h of IM-MFS-1. Moreover, the karyotype of each cell line is complex and has a number of important differences. For example, the cytogenetic region that contains the MET location on chromosome 7 has different aberrations, i.e., a gain in MUG-Myx1 and a loss in IM-MFS-1. Nevertheless, both cell lines maintain many features of the tissue of origin and represent good MFS models for preclinical studies. 

Okada et al. recently identified integrin-α10 as a driver of MFS progression through the activation of integrin-α10/TRIO/RICTOR signalling [28]. In our study, IM-MFS-1 continued to express stable levels of *ITGA10* up to passage 50. After a long time in culture (passage 100), the expression of this marker increases, contributing to the genetic alterations of the cancer cells in the in vitro microenvironment. 

*CTNNB1*, the gene encoding for β catenin, is overexpressed in several cancers [41]. Ogura et al. confirmed the upregulation of *CTNNB1* in MFS tissue and proposed this gene as a possible novel target [42]. In our study, *CTNNB1* expression increased constantly during the time in culture, confirming Ogura’s findings. β catenin is a mediator of the Wnt pathway and its nuclear accumulation is linked to cancer development [43]. Despite the high level of *CTNNB1* expression, IM-MFS-1 cells showed β catenin accumulation on the border of the cytoplasm. Further analysis is needed to investigate the status of the Wnt pathway in this culture. 

CD109 is a monomeric cell surface glycoprotein involved in the internalization and degradation of TGFβ. Emori et al. correlated the poor prognosis of MFS patients with the immunohistochemical overexpression of CD109 [30], and we previously showed that CD109 could represent an aid to the diagnosis of high-grade MFS [26]. Immunohistochemistry and PCR assays showed a high expression of CD109 in IM-MFS-1, highlighting the potential of CD109 as a new diagnostic and therapeutic target for MFS, and of IM-MFS-1 cell lines as a tool to better understand the role of CD109 in the biology of high-grade MFS. 

Sarcomas often have a complex karyotype profile [32,33]. Our results obtained from aCGH revealed the presence of gain and loss alterations in regions that contain genes involved in cancerogenesis, in particular oncogenes and tumor suppressors (Table 1). Moreover, alterations were detected in chromosome regions that are associated with MFS [32]. Choong et al. observed an association between 19p+ aberrations and unfavorable clinical outcome for MFS patients [34]. In our analysis, the detection of 19p+ alterations could match with the origin of IM-MFS-1 primary culture which was the product of the excision of a second local recurrence. These findings help to increase our knowledge of the karyotype profile of MFS cells and could be used (in the future) to identify specific karyotype alterations. 

The culture modifications also led to a change in sensitivity to chemotherapeutic drugs. Similar results were obtained by Bezdieniezhnykh et al. who compared the effect of different anticancer drugs on primary cultures and corresponding established cell lines [44]. We treated IM-MFS-1 cells with MFS therapies of first and second line options including trabectedin, a marine-derived anticancer agent [24,26,45]. The primary culture, obtained by sample digestion (passage 1), was not particularly affected by any of the treatments (Figure 5A). However, after six passages the culture showed an important increase in sensitivity to all of the treatments. Furthermore, the low activity of both drugs in the primary culture may have been due to the nature of the sample tested. In fact, after digestion the primary culture was composed of a heterogenic population of both malignant cells and stromal components of the patients’ tissue [46,47]. The presence of non-malignant cells contributed to reproducing the tumor microenvironment through the conservation of the crosstalk between cancer and healthy cells. In this context, the ex vivo microenvironment may influence the drug response, as shown by the low activity of all the treatments tested. The cancer cells seeded immediately after the patients’ surgery represented a more realistic sample for preclinical evaluations. The conservation of the phenotypic and genotypic features of the cancer mass were not altered by in vitro manipulation and partially contributed to the poor response to antiblastic treatments.

The composition of the immortalized cell line was characterized by cancer cells without contamination by other cells. Our model provides a high amount of biological material and is particularly suitable for the study of cancer processes. The acquisition of an immortalized phenotype inevitably resulted in the loss of some of the features of the patient’s tumor. Indeed, the prolonged time in an in vitro environment changed not only the response of the sample to drugs but also its genetic profile. As previously reported, the expression of several markers was modified during the culture period, attesting to the poor capacity of cell lines to reproduce the realistic features of human cancers.

IM-MFS-1 immortalization enabled us to monitor sensitivity and define the final response rate. From the first few passages onwards, the culture showed cytotoxic effects similar to those observed in clinical practice. As previously reported, EPI produced the greatest cytotoxic effect, as confirmed in the TUNEL assays, whereas trabectedin was less effective. Such results accurately reflect the efficacy of these treatments in a real-life clinical setting. Future in vivo analyses are needed to study the effect of the treatments used for MFS patients. 

In conclusion, we successfully isolated cancer cells from a surgical specimen of MFS, obtaining an immortalized cell line with no healthy cell components. We also compiled a phenotypic and genotypic profile of the cell line. Moreover, the achievement of a drug sensitivity plateau after a high number of passages provides an overview of the effects of the drugs tested. Further in-depth research is needed to study IM-MFS-1 tumorigenesis and drug sensitivity in in vivo models. An integrated approach between primary cultures and cell lines could be the best way to study MFS. The paucity of high-grade MFS cell lines also makes IM-MFS-1 a potentially useful tool for research purposes and the molecular and pharmacological characterization could make it an ideal candidate for bench-to-bedside studies.

## Figures and Tables

**Table 1:** Cytogenetic alterations on chromosome regions that contain candidate genes involved in cancer processes.

Cytoband	Gain/Loss	Size (kb ^1^)	Candidate Gene
1q25.2	Loss	327	*ABL2*
1p32.3	Gain	4756	*CDKN2C*
2q33.3	Loss	20	*CREB1*
3p13	Loss	639	*FOXP1*
5q22.2	Gain	304	*APC*
5q32	Gain	33	*PDGFRB*
6p22.3	Loss	2198	*SOX4*
7q31.2	Loss	520	*CAV1, MET*
7q34	Gain	150	*BRAF*
8q24.11-q24.13	Gain	5875	*EXT1*
8q24.21	Loss	430	*MYC*
10q11.22	Loss	61	*MAPK8*
10q23.2	Loss	224	*BMPR1A*
11q23.3	Loss	201	*SIK3*
12p12.1	Loss	534	*CASC1*
13q14.2	Loss	1238	*RB1*
18q11.2	Loss	2230	*SS18*
20q12-q13.11	Gain	4072	*MAFB*

^1^ kb, kilobase.

## Supplementary Materials

The following are available online, Figure S1: Relative quantification of genes related to migration and proliferation processes. Values are normalized to passage 6. Figure S2: Immunohistochemical staining for desmin, αSMA and S100 on IM-MFS-1 at passage 50. 20× magnification.
