# Supplementary material for: Characterization and Drug Sensitivity of a New High-Grade Myxofibrosarcoma Cell Line
Source: Cells. 2018 Oct 25;7(11):186. doi: 10.3390/cells7110186 (PMC6262427; doi:10.3390/cells7110186)
Supplement: Supplementary file 1 [file cells-07-00186-s001.pdf]

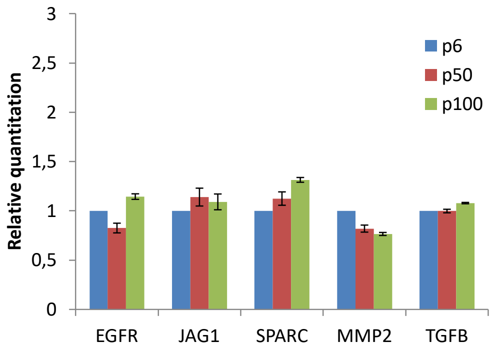

Supplementary Figure S1. Relative quantification of genes related to migration and proliferation processes. Values are normalized to passage 6.

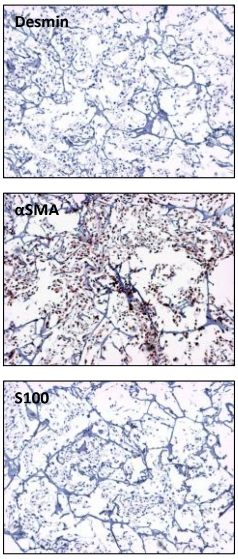

Supplementary Figure S2. Immunohistochemical staining for desmin, αSMA and S100 on IM-MFS-1 at passage 50. 20× magnification
